# Development and external validation of a nomogram for individualized adjuvant imatinib duration for high‐risk gastrointestinal stromal tumors: A multicenter retrospective cohort study

**DOI:** 10.1002/cam4.4673

**Published:** 2022-03-16

**Authors:** Ruolin Liu, Yingxin Wu, Jin Gong, Rui Zhao, Li Li, Qianyi Wan, Nan Lian, Xiaoding Shen, Lin Xia, Yuhou Shen, Haitao Xiao, Xiaoting Wu, Yi Chen, Ying Cen, Xuewen Xu

**Affiliations:** ^1^ Department of Burn and Plastic Surgery West China Hospital of Sichuan University Chengdu China; ^2^ Department of General Surgery, Center of Gastrointestinal and Minimally Invasive Surgery, The Third People's Hospital of Chengdu, Affiliated Hospital of Southwest Jiaotong University & The Second Affiliated Hospital of Chengdu Chongqing Medical University Chengdu China; ^3^ Research and Education Department Sichuan Friendship Hospital Chengdu China; ^4^ Department of Gastrointestinal Surgery West China Hospital of Sichuan University Chengdu China; ^5^ Laboratory of Mitochondrial and Metabolism West China Hospital of Sichuan University Chengdu China

**Keywords:** adjuvant imatinib, gastrointestinal stromal tumors, high risk, individualized treatment duration

## Abstract

**Introduction:**

The main emphasis of the research about adjuvant imatinib for high‐risk gastrointestinal stromal tumors (GISTs) is prolonging the treatment duration and ignores the heterogeneous that 10‐year recurrence rates ranged from about 20%–100%. Thus, this study evaluated the effect of different durations of adjuvant imatinib on outcomes in high‐risk GISTs to explore the feasibility of individual treatment.

**Methods:**

We analyzed 855 high‐risk GIST patients from three centers who underwent macroscopically complete resection between December 2007 and September 2020. The patients were divided into training (*n* =564) and two validation cohorts (*n* = 238 and53) based on their source. Recurrence‐free survival (RFS) was the primary point. Cox multivariate analysis was used to develop the nomogram. C‐index, time‐dependent area under the curves, and calibration plots were used to assess the performance of the nomogram.

**Results:**

Univariate analysis showed that longer adjuvant imatinib was significantly associated with better 5‐year RFS (*p* < 0.0001). Further investigation identified that the same high‐risk patients with lower tumor‐associated recurrence risk benefitted little from prolonged treatment and that the recommended adjuvant imatinib duration was insufficient for those with higher recurrence risk. A nomogram for predicting 2‐, 3‐, and 5‐year RFS based on different treatment durations and four major risk factors, namely, tumor site, size, mitotic count, and rupture status, was built and validated, with a C‐index of 0.82, 0.74, and 0.70 in training and two external validation cohorts, respectively. An online dynamic nomogram was further developed for clinical applications (https://ruolinliu666.shinyapps.io/GIST/), offering predictive recurrence rates based on different treatment durations and tumor features.

**Conclusions:**

We developed a nomogram to predict the recurrence risk for high‐risk patients according to tumor features and treatment durations of imatinib to help physicians on decision‐making for individualized treatment duration.

## Graphical abstract

We developed a nomogram to predict the recurrence risk for high‐risk patients according to the tumor features and the treatment duration of imatinib in this study, showing that prolonged adjuvant imatinib duration could not bring the optimum treatment effect to all high‐risk patients. Considering the great heterogeneity of recurrence probability among the high‐risk GISTs patients, the individualized adjuvant treatment duration aiming at personalized adjuvant treatment duration decision‐making should be valued and discussed.

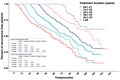

## 1 INTRODUCTION

Gastrointestinal stromal tumors (GISTs) are soft tissue malignancies of the gastrointestinal tract with a quite low incidence of 0.5 to 2 per 100,000, contributing to only 0.3%–0.5% for all gastrointestinal tumors.^1^, ^2^ Historically, patients with localized, primary GISTs undergoing complete resections have high recurrence rates and an average 5‐year recurrence rate of 40%–50%, due to poor response to conventional treatments (e.g., radiation and chemotherapy).^3^ In 2000, imatinib, a small‐molecule tyrosine kinase inhibitor (TKI), has truly changed the fate of patients with GISTs by not only revolutionizing the treatment strategy for locally advanced and metastatic GIST, but also controlling recurrence after complete resections. Thus, imatinib has been recommended as a first‐line agent for GIST treatment.^4^, ^5^

However, when imatinib is used to treat recurrent or unresectable GISTs, drug resistance will occur soon, leading to treatment failure. Adjuvant imatinib is the critical treatment measure to improve recurrence‐free survival (RFS) when administered after surgery to patients with high‐risk GISTs.^6^ Previous studies have clearly illustrated the benefits of adjuvant imatinib for high‐risk patients after surgery.^4^, ^7^, ^8^ Initially, two randomized clinical trials (RCTs), respectively demonstrated that 1 year of adjuvant imatinib can effectively reduce the recurrence rate of high‐risk GISTs (Z9001 ACOSOG) and that 2 years of treatment could prolong the RFS of patients with high recurrence rate compared with 1 year (EORTC 62024).^9^, ^10^, ^11^ Then, the RCT (SSG XVIII/AIO) suggested that 3 years of adjuvant imatinib effectively reduces the risk of recurrence by comparing the RFS of high‐risk patients with adjuvant imatinib for 12 and 36 months.^8^ Recently, the PERSIST‐5 trials and our retrospective study have discovered that 5 years of adjuvant imatinib therapy is tolerable and effective in patients with resected primary GISTs.^12^, ^13^ Interestingly, the results from these continuous studies suggested that a longer duration of adjuvant imatinib may be efficacious in high‐risk patients with resected primary GISTs. Based on the contour map, the 10‐year recurrence rates of high‐risk patients with complete resection ranged from about 20%–100%, indicating that the recurrence rate of the same high‐risk patients is heterogeneous.^14^ The main emphasis of the research on adjuvant imatinib for high‐risk GISTs is prolonging treatment duration and ignoring heterogeneous result. Thus, should the time of adjuvant imatinib be consistent or individualized treatment benefit the patients more, although the recurrence risk of patients are the same high‐risk patients?

This study aimed to analyze the effects of different durations of adjuvant imatinib on RFS for high‐risk GIST patients with different clinical characteristics. This study was conducted to build a predictive model to predict the recurrence probability for high‐risk GISTs based on their clinical features and adjuvant imatinib duration. Results will help doctors choose the most optimal imatinib treatment duration based on individuals' clinical characteristics.

## 2 METHODS

### 2.1 Study cohort

**FIGURE 1:**
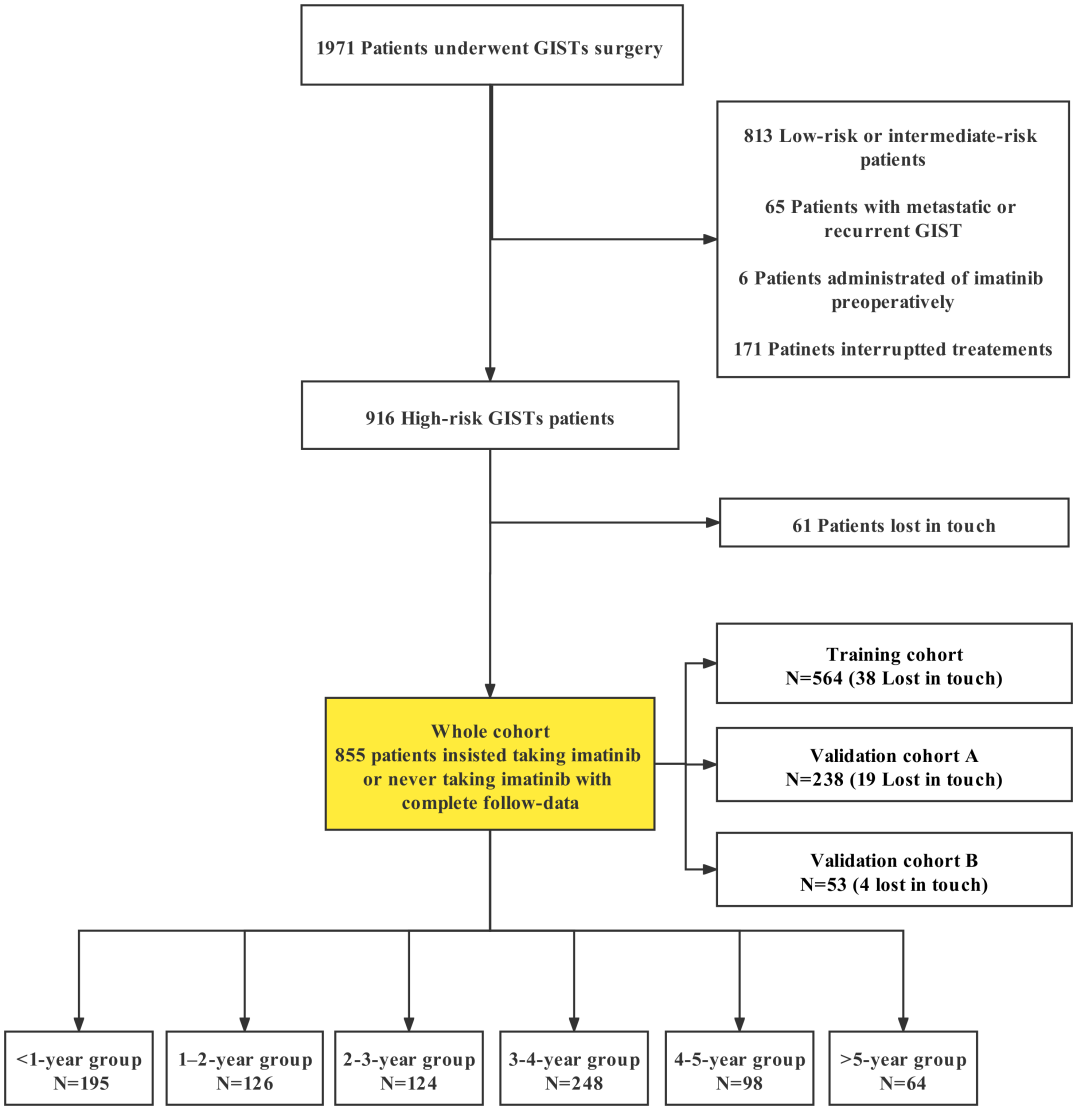
Flow of patients in the study. GIST, gastrointestinal stromal tumors

Data were retrospectively collected from 1971 patients with primary resectable c‐KIT‐positive GISTs in September 2020 from December 2007 at West China Hospital of Sichuan University, from December 2009 at Third People's Hospital of Chengdu, and from January 2015 at Sichuan Friendship Hospital (Figure 1). Patients were included if they (1) had undergone tumor resection as primary treatment; (2) had not received any TKI therapy (imatinib or sunitinib) before surgery; (3) had no evidence of metastatic GIST before or at surgery and no evidence of residual macroscopic disease after resection; (4) and had high recurrence risk as assessed by the physician based on the modified NIH criteria.^15^ In particular, this study aimed to clarify the effect of continuous adjuvant imatinib for patients with primary GISTs; the patients were also excluded if they retook imatinib after interrupting treatments for intolerance or side effects. Thus, the duration of adjuvant imatinib only included the period when patients received continuous adjuvant imatinib. By March 2021, we lost in touch with 61 high‐risk patients (Figure 1).

Finally, 855 patients were enrolled in this study (Figure 1); of which, 44 patients never received imatinib after resection for economic reasons and151 patients did not take imatinib for 12 months because of intolerance or severe side effects, which left 660 patients who could tolerate 400 mg of imatinib every day for more than 1 year. By design, the whole cohort was then divided into a training cohort and two validation cohorts for model establishment (Figure 1). The training cohort was from West China Hospital of Sichuan University, and the external validation cohorts were from another two institutions. During the construction, validation, and reporting of the clinical prediction model, transparent reporting of a multivariable prediction model for individual prognosis or diagnosis (TRIPOD) standards for multivariable prediction models were implemented.^16^ This study was performed in accordance with the ethical standards of the Helsinki Declaration and local regulations. The study was approved by the institutional review board of each institution and informed consent was obtained from all individuals. This study was registered in Chinese clinical trial registry (No. ChiCTR2100049423).

### 2.2 Design

At the beginning, all patients were divided into six groups according to the duration of adjuvant imatinib treatment for univariate analysis (Figure 1): <1‐year group (0–11 months, *n* = 195), 1–2‐year group (12–23 months, *n* = 126), 2–3‐year group (24–35 months, *n* = 124), 3–4‐year group (36–47 months, *n* = 248), 4–5‐year group (48–59 months, *n* = 98), and >5‐year group (more than 59 months, *n* = 64). The primary outcome was RFS, which was measured from the date of surgery to the date of GIST relapse or death, whichever occurred first, censoring patients alive without relapse or death at the date of the last follow‐up. Meanwhile, tumor mitosis, tumor size, tumor site, and tumor ruptured were factors affecting the recurrence risk of GISTs.^14^

### 2.3 Follow‐up procedures

Postoperative patients were followed up via telephone interview or outpatient service by specially trained researchers once every 1 to 3 months within 2 years and once every 3 to 6 months in the 3–5‐year period after surgery. Chest x‐ray or contrast‐enhanced computed tomography (CT) of the chest and magnetic resonance imaging (MRI) or CT of the pelvis and abdomen were tested with the first dose of imatinib. During the follow‐up, an MRI or CT of the pelvis and abdomen was performed at 3–6‐month intervals. Blood cell counts and chemistries were required every 1–3 months during the treatment period and subsequently at 6‐month intervals after imatinib discontinuation.

### 2.4 Statistical analyses

All statistical analyses were performed using R (version 4.0.3). Chi‐square test or Fisher's exact test was used to compare categorical data. Mann–Whitney *U* rank sum test or analysis of variance (ANOVA) was used to compare continuous data. Kaplan–Meier method was used to estimate RFS, and the differences in the survival curves among various subgroups were detected using the log‐rank test. Multivariate Cox regression was conducted to establish a prognostic risk model for predicting the RFS of high‐risk GIST patients. A nomogram was built to predict 2‐, 3‐, and 5‐year RFS by using the “rms” R package.^17^ We performed a time‐dependent receiver operating characteristic (ROC) curve via the “timeROC” package to evaluate the model's accuracy.^18^ The performance of the nomogram was assessed using the C‐index, area under the ROC curves (AUC), and calibration curve in the training and validation cohorts, and bootstraps with 1000 replicates were applied for internal validation of the established nomogram. A dynamic nomogram was developed and published by the “DynNom,” “rsconnect,” and “packrat” R package.^19^, ^20^, ^21^ All statistical tests were two‐sided, and the *p* < 0.05 was considered statistically significant.

## 3 RESULTS

### 3.1 Characteristics

A total of 855 patients, including 492 males and 363 females, met the inclusion criteria in the entire cohort. The mean duration of follow‐up, calculated from the data collection closure (March 2021), was 66 months (interquartile range, 43–90 months). The mean age was 54.50 ± 12.83 years. Four hundred and forty‐three (51.8%) of these patients had gastric GISTs, and the others had GISTs located at the small intestine (34.4%), colorectal (8.5%), or other sites (5.3%). The mean maximal tumor diameter was 9.92 ± 5.59 cm, and 129 (15.1%) patients had tumor larger than 15 cm, but with no evidence of microscopic disease at the margins and metastatic tumor at the chest and abdomen CT. The mean number of mitotic counts was 13 ± 18 per 50 high‐power fields (HPFs). Meanwhile, tumor rupture was recorded in 62 patients (7.3%). Among the 590 patients who had genetic analysis, the most common gene mutation was *KIT* exon 11 mutation (73.1%), followed by *PDGFRA* exon 18 mutation (8.8%), *KIT* exon nine mutation (8.0%), *KIT* exon 13 mutation (3.1%), and *PDGFRA* exon 12 mutation (2.5%). The baseline characteristics of the patients were generally balanced between the groups (Table 1).

**TABLE 1:** Main patient characteristics in different treatment durations

Characteristics	Overall	Treatment duration (years)						*p* value
		0 ≤ *t* <1	1 ≤ *t* <2	2 ≤ *t* <3	3 ≤ *t* <4	4 ≤ *t* <5	*t* ≥ 5	
*n*	855	195	126	124	248	98	64	
Gender (%)								0.803
Male	492 (57.5)	114 (58.5)	75 (59.5)	71 (57.3)	147 (59.3)	52 (53.1)	33 (51.6)	
Female	363 (42.5)	81 (41.5)	51 (40.5)	53 (42.7)	101 (40.7)	46 (46.9)	31 (48.4)	
Age (mean ± SD)	54.50 (12.83)	55.27 (13.83)	54.08 (12.92)	55.77 (14.22)	53.04 (12.45)	55.27 (10.61)	54.97 (10.95)	0.339
Size (cm) (mean ± SD)	9.92 (5.59)	10.68 (5.75)	9.52 (5.21)	8.92 (4.76)	10.02 (5.85)	10.14 (6.28)	9.66 (4.92)	0.124
Size (cm) (%)								
2.1–5.0	140 (16.4)	24 (12.3)	22 (17.5)	21 (16.9)	49 (19.8)	15 (15.3)	9 (14.1)	
5.1–10.0	448 (52.4)	96 (49.2)	64 (50.8)	78 (62.9)	118 (47.6)	58 (59.2)	34 (53.1)	
10.1–15.0	138 (16.1)	40 (20.5)	26 (20.6)	10 (8.1)	39 (15.7)	12 (12.2)	11 (17.2)	
>15.0	129 (15.1)	35 (17.9)	14 (11.1)	15 (12.1)	42 (16.9)	13 (13.3)	10 (15.6)	
Site (%)								0.259
Gastric	443 (51.8)	108 (55.4)	71 (56.3)	59 (47.6)	123 (49.6)	55 (56.1)	27 (42.2)	
Non‐gastric	412 (48.2)	87 (44.6)	55 (43.7)	65 (52.4)	125 (50.4)	43 (43.9)	37 (57.8)	
Ruptured (%)								0.274
Yes	62 (7.3)	14 (7.2)	13 (10.3)	7 (5.6)	13 (5.2)	7 (7.1)	8 (12.5)	
No	793 (92.7)	181 (92.8)	113 (89.7)	117 (94.4)	235 (94.8)	91 (92.9)	56 (87.5)	
Mitotic/50 HPFs (mean ± SD)	13.85 (18.52)	13.73 (26.18)	12.67 (7.61)	16.17 (24.27)	13.33 (12.96)	13.07 (14.11)	15.28 (17.08)	0.668
Mitotic/50 HPFs (%)								
≤5	108 (12.6)	36 (18.5)	13 (10.3)	21 (16.9)	20 (8.1)	9 (9.2)	9 (14.1)	
6–10	335 (39.2)	73 (37.4)	40 (31.7)	46 (37.1)	106 (42.7)	45 (45.9)	25 (39.1)	
11–20	332 (38.8)	70 (35.9)	58 (46.0)	46 (37.1)	103 (41.5)	32 (32.7)	23 (35.9)	
>20	80 (9.4)	16 (8.2)	15 (11.9)	11 (8.9)	19 (7.7)	12 (12.2)	7 (10.9)	
Mutated exon (%)								0.122
*KIT* exon 11	431 (73.1)	95 (70.9)	62 (68.9)	66 (72.5)	118 (76.6)	51 (73.9)	39 (75.0)	
*KIT* exon 13	18 (3.1)	3 (2.2)	3 (3.3)	4 (4.4)	3 (1.9)	4 (5.8)	1 (1.9)	
*KIT* exon 9	47 (8.0)	7 (5.2)	7 (7.8)	7 (7.7)	12 (7.8)	7 (10.1)	7 (13.5)	
*PDGFRA* exon 12	15 (2.5)	3 (2.2)	3 (3.3)	4 (4.4)	3 (1.9)	2 (2.9)	0 (0.0)	
*PDGFRA* exon 18	52 (8.8)	11 (8.2)	12 (13.3)	6 (6.6)	16 (10.4)	4 (5.8)	3 (5.8)	
Wild type	27 (4.6)	15 (11.2)	3 (3.3)	4 (4.4)	2 (1.3)	1 (1.4)	2 (3.8)	

Abbreviations: HPF, high‐power fields; SD, standard deviation.

### 3.2 Longer adjuvant imatinib duration contributed to more favorable RFS

**FIGURE 2:**
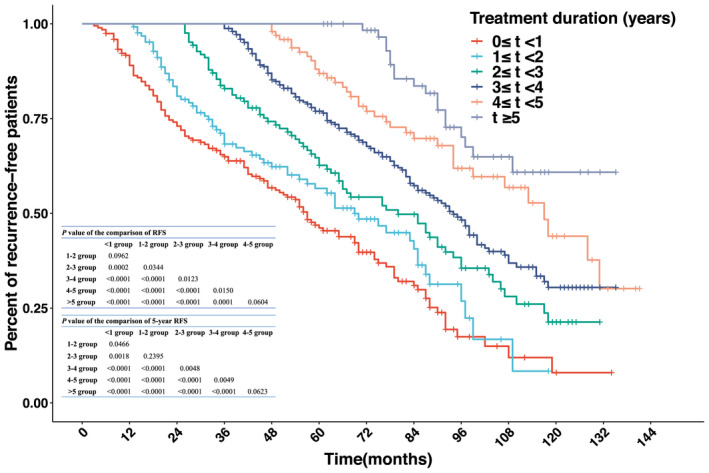
Kaplan–Meier estimates of the recurrence‐free survival of 855 patients in different imatinib treatment duration groups. RFS, recurrence‐free survival

Kaplan–Meier method and log‐rank test model were used to analyze the effect of the adjuvant imatinib duration on RFS in different groups. The median RFS among all patients was 87 (95% CI, 83–92) months. The 2‐, 3‐, and 5‐year RFS rates of all patients were 91.2%, 84.9%, and 68.3%, respectively. The median RFS of <1‐year group, 1–2‐year group, 2–3‐year group, 3–4‐year group, and 4–5‐year group were 57 (95% CI, 47–70) months, 69 (95% CI, 57–85) months, 80 (95% CI, 66–94) months, 94 (95% CI, 85–102) months, and 117 (95% CI, 99–not reached, NR) months, respectively, and the median RFS of >5‐year group was not reached (*p* < 0.0001, Figure 2). Interestingly, no significant differences in RFS were found between 1 and 2‐year group and <1‐year group (*p* = 0.0962) or between >5‐year group and 4–5‐year group (*p* = 0.0604), while the RFS between other groups had significant differences (Figure 2). Therefore, the RFS of the >5‐year group and 4–5‐year group was the longest, and the 3–4‐year group and 2–3‐year group was the second, while the worst groups of RFS were the 1–2‐year group and <1‐year group. Most recurrences of primary GISTs arose within the first 5 years after surgery; thus, the 5‐year RFS was an important outcome for assessing therapeutic effects. Hence, our data showed that the 5‐year RFS rates in <1‐year group, 1–2‐year group, 2–3‐year group, 3–4‐year group, 4–5‐year group, and >5‐year group were 46.2%, 56.6%, 62.7%, 77.0%, 86.9%, and 100.0%, respectively (*p* <0.0001, Figure 2). In other words, for one extension year of adjuvant treatment, the 5‐year RFS rate was improved by about 10.8%. Furthermore, the results of the 5‐year RFS rate among the six groups were consistent with the results of RFS, indicating no significant difference in the 5‐year RFS between 1–2‐year group and 2–3‐year group (*p* = 0.2395) or between >5‐year group and 4–5‐year group (*p* = 0.0623); the 5‐year RFS between the other groups had significant differences (Figure 2). The above analysis of RFS revealed that the longer the treatment duration lasted, the more favorable the RFS was, which was also indirectly supported by previous studies.^8^, ^13^, ^22^, ^23^ Moreover, in the same high‐risk patients, the tumor size, site, rupture, and mitosis count, all of which were associated with RFS, were of great diversity. Thus, we further analyzed the impact of these risk factors on RFS.

### 3.3 Part of risk factors still resulted in the deterioration of RFS despite the lack of difference in treatment duration

Univariate analysis was applied to discuss the effect of tumor size, site, rupture, and mitosis count on RFS (Figure S1). Based on research from Joensuu's team and Miettinen's team, respectively about recurrence risk assessment of GISTs,^14^, ^24^ tumor size was divided into four groups according to the maximum tumor diameter; the tumor site had two groups, namely, gastric and non‐gastric; tumor rupture was determined by surgical records, and tumor mitosis was classified into four groups by average count in 50 HPFs of three pathologists. The results revealed that tumor size, rupture, and mitosis count, not including tumor site, were significantly associated with RFS (Figure S1).

First, we analyzed the RFS between different tumor size groups, showing that tumor >15.0 cm was the most unfavorable outcome; differences in RFS among the three other groups, 2.0–5.0, 5.1–10, and 10.1–15.0 cm, were not significant (Figure S1A). Besides, the patients in the four sizes group received adjuvant treatment at the same duration (Table S1), demonstrating inadequate adjuvant imatinib duration for the patients with tumor size >15.0 cm. Similarly, the tumor rupture also led to poor RFS (Figure S1B), though no significant difference in treatment duration was observed (Table S2), indicating that prolonging the adjuvant duration was necessary for patients with tumor rupture. Moreover, the univariate analysis of tumor mitosis about RFS was in accordance with that of tumor size and rupture (Figure S1C and Table S3). Tumor mitosis had the most significant impact on RFS even though adjuvant imatinib durations of the four groups were similar. By contrast, the adjuvant treatment durations of patients with gastric and non‐gastric GISTs were identical (Table S4); meanwhile, the times of RFS between them were not significantly different (Figure S1D). It illustrated that sufficient adjuvant imatinib duration could eliminate the adverse effect of non‐gastric GISTs on prognosis. The analysis of these risk factors showed that high‐risk patients with tumor >15 cm, ruptured tumor, or high tumor mitosis should take adjuvant imatinib for longer duration than high‐risk patients with tumor <15 cm, unruptured tumor, or low tumor mitosis, indicating that individualized treatment should be considered for the same high‐risk patients.

### 3.4 Cox multivariate regression and nomogram were performed and validated to discuss the relationship between five factors and RFS

**FIGURE 3:**
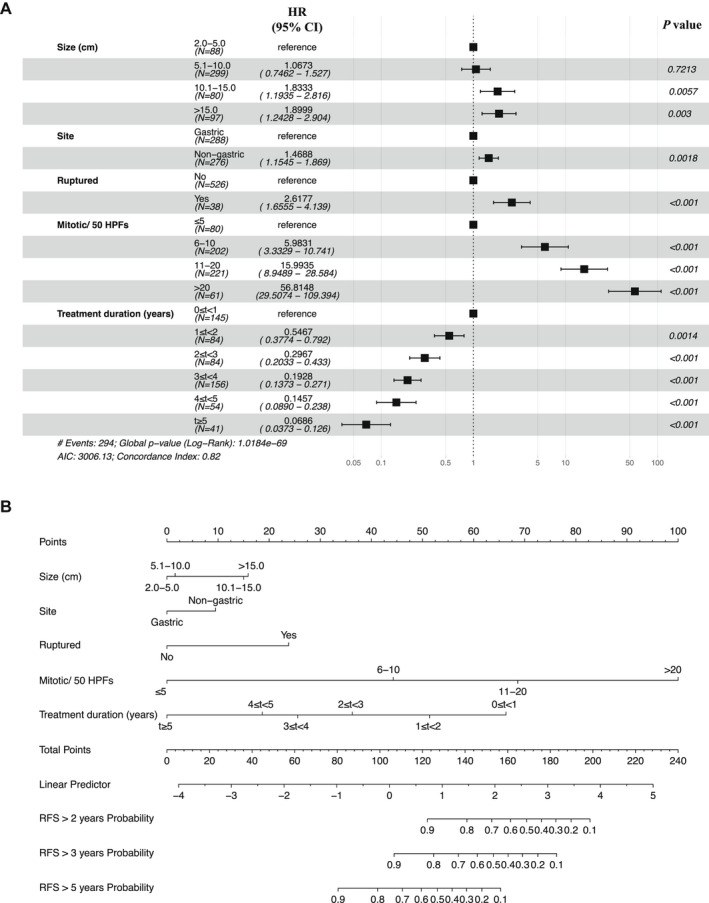
Forest plot (A) of multivariate Cox regression analysis and nomogram (B) for predicting 2‐, 3‐, and 5‐year recurrence‐free survival. CI, confidence interval; HPF, high‐power fields; HR, hazard ratio; RFS, recurrence‐free survival

To better explore the co‐effect of tumor size, site, mitotic count, rupture status, and imatinib adjuvant treatment duration on RFS, Cox multivariate regression analysis was conducted for the five factors in the training cohort, from West China Hospital of Sichuan University (Table 2); the results indicated that they were independently associated with RFS (Figure 3A). Furthermore, we established a nomogram to predict 2‐, 3‐, and 5‐year RFS based on the multivariate analyses, which could help us understand the optimal treatment duration for the same high‐risk patients with different characteristics (Figure 3B). Nomogram could be understood as the graphic display for the model, in which points are assigned according to the rank order of the effect estimates. Factors assigned the highest number of points was tumor mitosis (>20/50 HPFs: HR 56.81, 95% CI 29.51–109.39; 11–20/50 HPFs: HR 15.99, 95% CI 8.95–28.58; 6–10/50 HPFs: HR 5.98, 95% CI 3.33–10.74; ≤5/50 HPFs as reference), followed by tumor rupture, tumor size, and tumor site. Adjuvant imatinib duration was the only protective factor for the RFS of GISTs, in which longer treatment duration contributed to lower points (Figure 3B).

**TABLE 2:** Tumor features and imatinib treatment duration in different cohorts

	Training cohort	Validation cohort A	Validation cohort B
*n*	564	238	53
Size (cm) (mean [SD])	10.16 (5.93)	9.59 (4.96)	8.92 (4.21)
Size (cm) (%)			
2.0–5.0	88 (15.6)	42 (17.6)	10 (18.9)
5.1–10.0	299 (53.0)	117 (49.2)	32 (60.4)
10.1–15.0	80 (14.2)	49 (20.6)	9 (17.0)
>15.0	97 (17.2)	30 (12.6)	2 (3.8)
Site (%)			
Gastric	288 (51.1)	131 (55.0)	24 (45.3)
Non‐gastric	276 (48.9)	107 (45.0)	29 (54.7)
Ruptured (%)			
Yes	38 (6.7)	20 (8.4)	4 (7.5)
No	526 (93.3)	218 (91.6)	49 (92.5)
Mitotic/50 HPFs (mean [SD])	14.79 (21.90)	12.61 (9.19)	9.53 (4.39)
Mitotic/50 HPFs (%)			
≤5	80 (14.2)	24 (10.1)	4 (7.5)
6–10	202 (35.8)	103 (43.3)	30 (56.6)
11–20	221 (39.2)	93 (39.1)	18 (34.0)
>20	61 (10.8)	18 (7.6)	1 (1.9)
Treatment duration (years) (%)			
0 ≤ *t* <1	145 (25.7)	43 (18.1)	7 (13.2)
1 ≤ *t* <2	84 (14.9)	31 (13.0)	11 (20.8)
2 ≤ *t* <3	84 (14.9)	28 (11.8)	12 (22.6)
3 ≤ *t* <4	156 (27.7)	77 (32.4)	15 (28.3)
4 ≤ *t* <5	54 (9.6)	38 (16.0)	6 (11.3)
*t* ≥ 5	41 (7.3)	21 (8.8)	2 (3.8)

Abbreviations: HPF, high‐power fields; SD, standard deviation.

**FIGURE 4:**
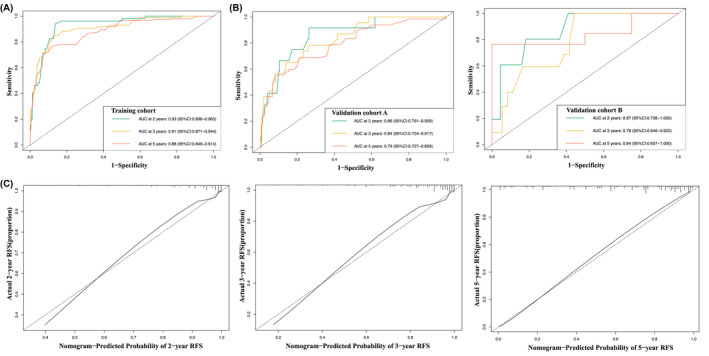
The AUC curve of the nomogram for predicting 2‐, 3‐, and 5‐year recurrence‐free survival in the training cohort and (A) and validation cohorts (B) and calibration curve in training cohort (C). The *x*‐axis depicts the probability of RFS using the nomogram, and the *y*‐axis depicts the actual rate of RFS. The solid line denotes the nomogram's apparent prediction, and the dashed line indicates the ideal estimation. AUC, area under the curves; RFS, recurrence‐free survival

The analysis of AUC, C‐index, and calibration curve was performed in the training cohort, external validation cohort A (from Third People's Hospital of Chengdu), and external validation cohort B (from Sichuan Friendship Hospital) to evaluate the performance of the constructed nomogram (Table 2). The AUC of the nomogram for 2‐, 3‐, and 5‐year RFS prediction were 0.93, 0.91, and 0.88 in the training cohort; 0.86, 0.84, and 0.79 in validation cohort A; 0.87, 0.78, and 0.84 in validation cohort B (Figure 4A,B). The C‐index of the nomogram was 0.82, 0.74, and 0.70 in the training cohort, validation cohort A, and validation cohort B, which proved its outstanding prediction accuracy. Furthermore, bootstrap method with 1000 replicates was used for internal validation of the developed nomogram, indicating that the validated AUCs of the nomogram for 2‐, 3‐, and 5‐year RFS prediction were 0.92, 0.89, and 0.87, respectively, and the inner validated C‐index was 0.81. Furthermore, the calibration curves showed good consistency between the prediction results by nomogram and actual observation for 2‐, 3‐, and 5‐year RFS predictions in the training and validation cohorts (Figure 4C and Figure S2). The results revealed that the nomogram established had a relatively high discrimination level and calibration performance for predicting the RFS of high‐risk patients treated with adjuvant imatinib.

### 3.5 Dynamic nomogram benefited the individualized adjuvant imatinib duration decision‐making

A dynamic nomogram was developed to further enhance the usefulness of our model and could be found at the website of https://ruolinliu666.shinyapps.io/GIST/. When using this website, the physician or patient just need to choose tumor size, tumor mitotic count, ruptured tumor status, and expected adjuvant imatinib duration, the recurrence probability plot would show, which could be able to find the most optimum adjuvant imatinib time for the same high‐risk patient with different characteristics.

Through the dynamic nomogram, patients with lower recurrence risk factors, including small tumor size, no rupture, gastric site, and low tumor mitotic count, benefitted little from prolonged treatment; for those with higher risk factors such as a large tumor, rupture, non‐gastric tumor, and high tumor mitotic count, the recommended adjuvant imatinib duration (3 years in most guidelines) was not sufficient. For example, for a patient with a tumor size of 12 cm, mitosis count of 3/50HPFs, and unruptured stomach GIST, the 5‐year recurrence rate was 3.2% (95% CI: 0.9%–5.4%) if treated with imatinib for 2–3 years, which was much lower than the 5‐year recurrence rate of medium‐risk patients with primary GISTs. If prolonging the treatment duration to 5 years, then the 5‐year recurrence rate of the patient would be 0.7% (95% CI: 0.1%–1.4%), which could not significantly benefit the patient. On the other hand, if prolonging the treatment duration from 2–3 years to more than 5 years for a patient with a tumor size of 30 cm, the mitosis count of 15/50HPFs ruptured intestinal GIST, then the 5‐year recurrence rate would be decreased from 87.0% (95% CI: 62.0%–96.0%) to 38.0% (95% CI: 12.6%–56.0%). Therefore, the main emphasis of research about adjuvant imatinib for high‐risk gastrointestinal stromal tumors should shift from prolonged adjuvant duration to individualized treatment.

## 4 DISCUSSION

Complete resection and adjuvant TKI therapy are the standard treatments for high recurrence risk of primary GISTs.^8^, ^13^, ^23^ The four following TKIs have been approved for the management of advanced GISTs: imatinib, sunitinib, regorafenib, and avapritinib; imatinib is usually the best tolerated of the four and the standard first‐line treatment for adjuvant therapy.^25^, ^26^ Continuous imatinib therapy over an extended period is more effective in controlling tumors.^27^, ^28^ Meanwhile, previous studies demonstrated that for advanced GIST, interruption of imatinib treatment led to rapid disease progression and resistance, while long‐term continuous imatinib therapy reduced the risk of disease progression.^27^, ^28^, ^29^, ^30^ Even regaining tumor control, tumors did not achieve an equal level of response compared with the best response before interruption.^31^ Therefore, the appropriate duration of continuous imatinib treatment is crucial in prolonging the RFS and preventing the resistance of high‐risk patients with primary GISTs.

However, patient compliance is an uncertain factor in clinical practice. In this study, 855 high‐risk patients who received continuous adjuvant imatinib or did not receive imatinib for economic reasons after complete resection were retrospectively collected, in whom the longest follow‐up time was 141 months. Compared with the RCTs,^8^, ^23^ the most distinctive advantage of our study was a Real‐World Research (RWR), in which the patients' therapeutic schedule was determined not only by the severity of the disease but also by other factors such as expensive costs or the side effect of imatinib. Therefore, the duration of adjuvant imatinib in this study was much more diversified than that in RCTs, which provided more abundant data to benefit from a more accurate analysis of the relationship between the duration of adjuvant imatinib and RFS of patients. Besides, our data allowed for a more detailed investigation of RFS in different high‐risk patients receiving different durations of continuous adjuvant imatinib.

Among 855 patients, 17.7% (n = 151) of the patients discontinued adjuvant imatinib for intolerance or severe side effects within 1 year, which was more than that in the RCT including Westerners only.^13^ Thus, in Asia, low‐dose continuous adjuvant imatinib should be recommended for intolerable patients, as supported by relevant research.^32^, ^33^ As the recommended dose cannot be tolerated for some Asian patients, finding the optimal treatment duration could be more important than increasing the dose of imatinib for such patients. Therefore, seeking the appropriate adjuvant duration for imatinib might be the key point of studies at this stage. The results from Figure 2 demonstrates that the prolonged adjuvant imatinib improved the RFS of the patients. The SSG XVIII/AIO trial showed that 5‐year RFS rates of adjuvant imatinib for 1 and 3 years were 48% and 66%, respectively.^8^ The EORTC 62024 trial reported that the 5‐year RFS rate of the patients with adjuvant imatinib for 2 years was 69%.^23^ Meanwhile, the 5‐year RFS rate of the patients who received adjuvant imatinib for 4–5 years in our study was 86.9%, which was higher than that in the two trials. The PERSIST‐5 trial showed that 5‐year RFS rate was above 90% in the adjuvant imatinib for 5 years, which resembled our result.^13^ Based on previous reports and our retrospective data, adjuvant imatinib for 5 years might further decrease recurrence than 3 years but was still not recommended strongly.

The results highlight whether it is profitable for all patients to keep extending the duration of adjuvant imatinib. In previous research, the 10‐year recurrence rate of the same high‐risk groups was extraordinarily heterogeneous, ranging from about 20%–100%.^14^ Similarly, tumor size, rupture, and mitosis count still influenced the prognosis even though the patients were of the same high‐risk level and received adjuvant imatinib (Figure S1). Therefore, not all high‐risk patients would obtain the optimal treatment effect by prolonging adjuvant imatinib on account of the heterogeneous result in patients with the same recurrence risk of GISTs. In other words, overextending adjuvant imatinib in high‐risk GISTs with small, unruptured tumor, gastric site, and low tumor mitotic count is unreasonable. Moreover, the duration of adjuvant imatinib for patients with large, ruptured, non‐gastric tumor, and high mitotic count was still insufficient. Thus, the individualized duration of adjuvant imatinib should be considered for the same high‐risk patients with different tumor sizes, sites, rupture status, and mitotic counts. The definition of high‐risk GISTs should be more precise, and a previous study suggested that the classification of very high risk had been conceived for guiding the treatment.^34^ To achieve the purpose of individualized treatment, we evaluated the score of risk factors and the duration of adjuvant imatinib and then established an evaluation system, a nomogram. The nomogram transformed complex regression equations into visual graphs, making the results of the predictive models more readable and facilitating patient assessment. Through the analyses, tumor mitotic count has the most significant effect on the recurrence of high‐risk GISTs, and tumor site had the lowest effect. Thus, 5‐year adjuvant imatinib or more should be recommended for high‐risk patients with high tumor mitotic count. Future studies on adjuvant imatinib treatment for GISTs should focus on personalized treatment duration. At the same time, the risk classification of patients with GISTs should be further refined, especially for high‐risk patients. The treatment duration of imatinib can be better determined according to the refined classification criteria.

Although the nomogram can point out which high‐risk patients should take adjuvant imatinib longer, it is still not practical in achieving individualized treatment. For practical reason, we designed an online tool, which was implemented by a dynamic nomogram. When using this website, the tumor size, mitotic count, rupture status, site, and duration of adjuvant imatinib are just inputted, and the recurrence plot would be calculated. Therefore, by only entering the expected adjuvant imatinib duration, the percentage rate of the recurrence risk reduced by adjuvant imatinib would be calculated accurately. The predicted recurrence probability for high‐risk patients in the online nomogram further verified the above conclusion that individualized treatment is of great necessity.

This study has several limitations. First, the major limitation is its retrospective nature. Second, as a multicenter study with a rather long study period, some of the genetic tests conducted in the patients were deficient, and we did not include the gene mutation for all patients. Therefore, well‐designed prospective research with a large sample size and complete genetic testing data are necessary to obtain more reliable evidence in the future. However, the study has strengths. We focused on patients with GIST and high risk of recurrence for whom postoperative imatinib therapy is highly essential. Furthermore, the model established had a relatively high discrimination level and calibration performance for predicting the RFS of high‐risk patients treated with adjuvant imatinib in the training and external validation cohorts. The online tool is easy to use in selecting the optimal imatinib treatment duration for a single high‐risk individual. Moreover, all the factors included in our model for predicting the RFS of the patients were easy to obtain in routine clinical practice, which further increased the practicality of our model.

## 5 CONCLUSIONS

We developed a nomogram to predict the recurrence risk of high‐risk patients according to tumor features and treatment duration of imatinib, showing that prolonged adjuvant imatinib duration could not bring the optimum treatment effect to all high‐risk patients. Considering the great heterogeneity of recurrence probability among high‐risk GIST patients, individualized adjuvant treatment duration aiming at personalized adjuvant treatment duration decision‐making should be valued and discussed.

## CONFLICT OF INTEREST

None.

## AUTHOR CONTRIBUTIONS

Ruolin Liu, Rui Zhao, Ying Cen, and Xuewen Xu designed this study; Wingxin Wu, Jin Gong, Rui Zhao, Qianyi Wan, Li Li, and Xiaoding Shen contributed to collect data; Ruolin Liu, Yingxin Wu, Jin Gong, and Yuhou Shen analyzed and interpreted the data; Ruolin Liu, Nan Lian, Lin Xia, and Haitao Xiao performed statistical analysis; Ruolin Liu, Yingxin Wu, Jin Gong, and Xiaoting Wu drafted the manuscript; Yi Chen, Ying Cen, and Xuewen Xu supervised this study and revised the manuscript. All authors read and approved the final manuscript.

## ETHICAL APPROVAL STATEMENT

This study was performed in accordance with the ethical standards of the Helsinki Declaration and local regulations. The study was approved by the institutional review board of West China Hospital of Sichuan University, Third People's Hospital of Chengdu, and Sichuan Friendship Hospital. Informed consent was obtained from all the individuals. This study was registered in Chinese clinical trial registry (No. ChiCTR2100049423).

## Supporting information


Figure S1

Figure S2

Table S1
Table S2
Table S3
Table S4

## Data Availability

The data presented in this study are available upon request from the corresponding author.
