# Supplementary figures and images for: Development and external validation of a nomogram for individualized adjuvant imatinib duration for high‐risk gastrointestinal stromal tumors: A multicenter retrospective cohort study
Source: Cancer Med. 2022 Mar 16;11(16):3093–105. doi: 10.1002/cam4.4673 (PMC9385591; doi:10.1002/cam4.4673)

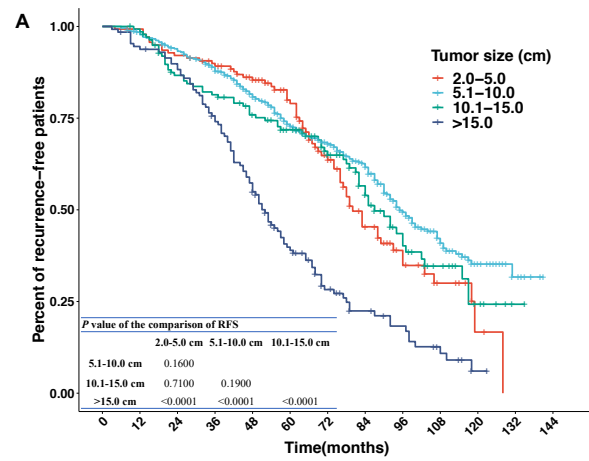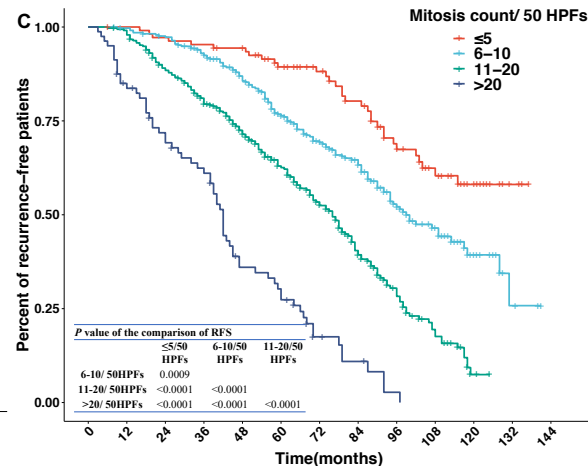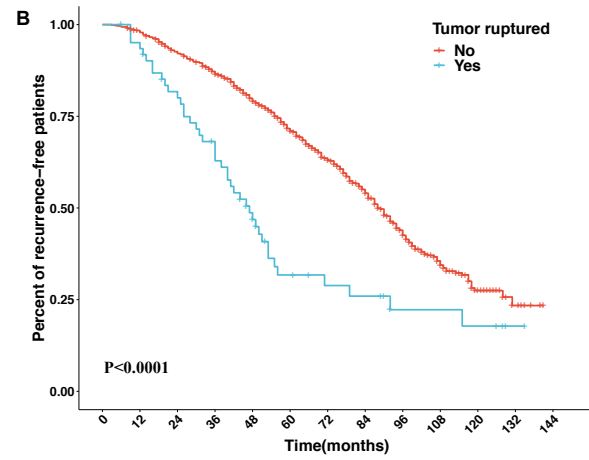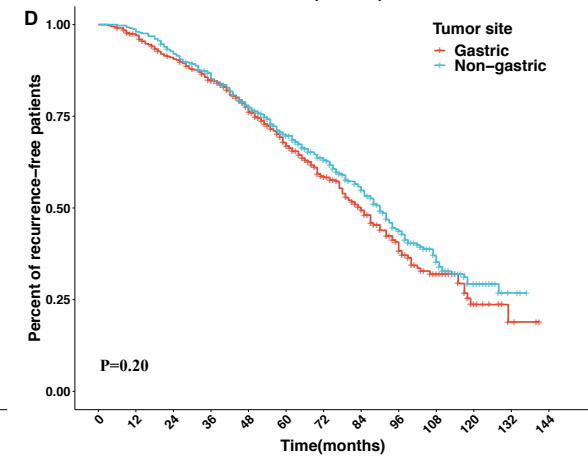

Supplement: Supplementary file 1 — Figure S1 [file CAM4-11-3093-s001.pdf]

## A Validation cohort A

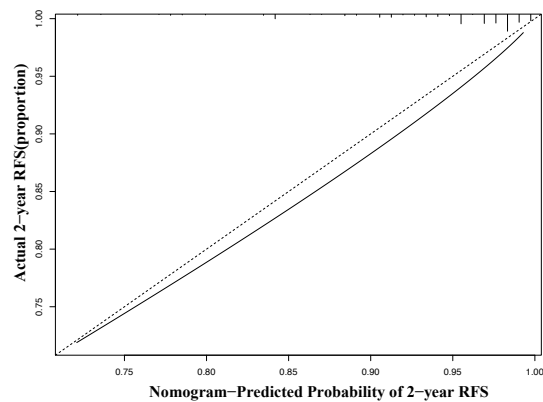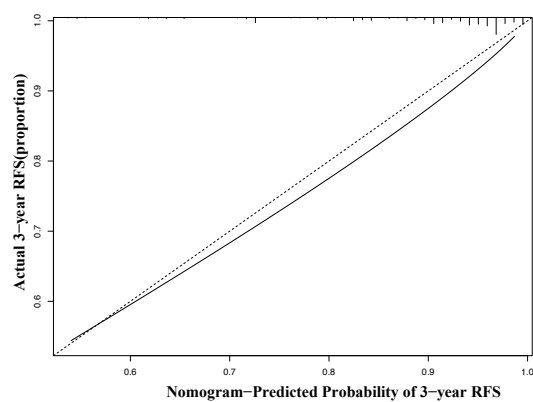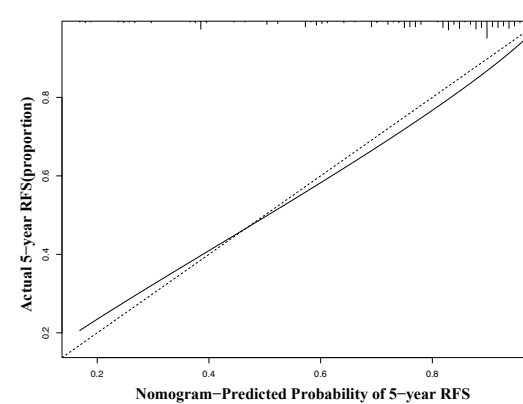

## B Validation cohort B

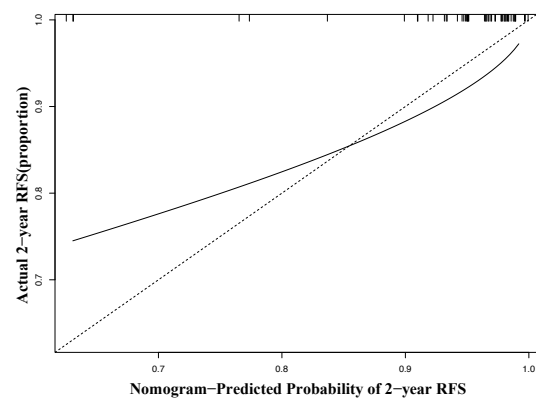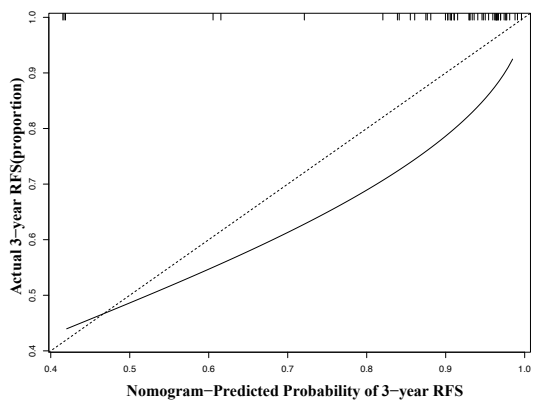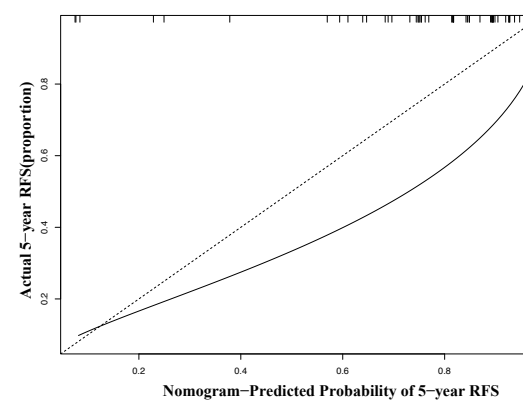

Supplement: Supplementary file 2 — Figure S2 [file CAM4-11-3093-s003.pdf]
