# Supplementary material for: Development and external validation of a nomogram for individualized adjuvant imatinib duration for high‐risk gastrointestinal stromal tumors: A multicenter retrospective cohort study
Source: Cancer Med. 2022 Mar 16;11(16):3093–105. doi: 10.1002/cam4.4673 (PMC9385591; doi:10.1002/cam4.4673)
Supplement: Supplementary file 3 — Table S1 Table S2 Table S3 Table S4 [file CAM4-11-3093-s002.docx]

**Supporting tables**

**Table S1.** The analysis of patient clinical characteristics based on tumor size.

| **Characteristics** | **Overall** | **Tumor size (cm)** | | | | ***P* value** |
| --- | --- | --- | --- | --- | --- | --- |
|  |  | **2.0-5.0** | **5.1-10.0** | **10.1-15.0** | **>15.0** |  |
| **n** | 855 | 140 | 448 | 138 | 129 |  |
| **Site** |  |  |  |  |  | 0.002 |
| Gastric | 443 (51.8) | 74 (52.9) | 206 (46.0) | 83 (60.1) | 80 (62.0) |  |
| Non-gastric | 412 (48.2) | 66 (47.1) | 242 (54.0) | 55 (39.9) | 49 (38.0) |  |
| **Mitotic/ 50 HPFs (mean±SD)** | 13.85 (18.52) | 13.96 (6.73) | 11.22 (10.95) | 12.30 (11.23) | 24.53 (39.29) | <0.001 |
| **Mitotic/ 50 HPFs (%)** |  |  |  |  |  |  |
| ≤5 | 108 (12.6) | 0 (0.0) | 65 (14.5) | 30 (21.7) | 13 (10.1) |  |
| 6-10 | 335 (39.2) | 32 (22.9) | 210 (46.9) | 49 (35.5) | 44 (34.1) |  |
| 11-20 | 332 (38.8) | 103 (73.6) | 146 (32.6) | 46 (33.3) | 37 (28.7) |  |
| >20 | 80 (9.4) | 5 (3.6) | 27 (6.0) | 13 (9.4) | 35 (27.1) |  |
| **Ruptured (%)** |  |  |  |  |  | <0.001 |
| Yes | 62 (7.3) | 0 (0.0) | 11 (2.5) | 13 (9.4) | 38 (29.5) |  |
| No | 793 (92.7) | 140 (100.0) | 437 (97.5) | 125 (90.6) | 91 (70.5) |  |
| **Treatment duration (years) (%)** | |  |  |  |  | 0.086 |
| 0≤t<1 | 195 (22.8) | 24 (17.1) | 96 (21.4) | 40 (29.0) | 35 (27.1) |  |
| 1≤t<2 | 126 (14.7) | 22 (15.7) | 64 (14.3) | 26 (18.8) | 14 (10.9) |  |
| 2≤t<3 | 124 (14.5) | 21 (15.0) | 78 (17.4) | 10 (7.2) | 15 (11.6) |  |
| 3≤t<4 | 248 (29.0) | 49 (35.0) | 118 (26.3) | 39 (28.3) | 42 (32.6) |  |
| 4≤t<5 | 98 (11.5) | 15 (10.7) | 58 (12.9) | 12 (8.7) | 13 (10.1) |  |
| t≥5 | 64 (7.5) | 9 (6.4) | 34 (7.6) | 11 (8.0) | 10 (7.8) |  |

*SD*, Standard Deviation; *HPF*, High-Power Fields

**Table S2.** The analysis of patient clinical characteristics based on tumor rupture.

| **Characteristics** | **Overall** | **Ruptured** | | ***P* value** |
| --- | --- | --- | --- | --- |
|  |  | **No** | **Yes** |  |
| **n** | 855 | 793 | 62 |  |
| **Size (cm) (mean±SD)** | 9.92 (5.59) | 9.36 (5.12) | 17.19 (6.23) | <0.001 |
| **Size (cm) (%)** |  |  |  |  |
| 2.0-5.0 | 140 (16.4) | 140 (17.7) | 0 (0.0) |  |
| 5.1-10.0 | 448 (52.4) | 437 (55.1) | 11 (17.7) |  |
| 10.1-15.0 | 138 (16.1) | 125 (15.8) | 13 (21.0) |  |
| >15.0 | 129 (15.1) | 91 (11.5) | 38 (61.3) |  |
| **Mitotic/50 HPFs (mean±SD)** | 13.85 (18.52) | 13.30 (14.43) | 20.97 (45.20) | 0.002 |
| **Mitotic/50 HPFs (%)** |  |  |  |  |
| ≤5 | 108 (12.6) | 93 (11.7) | 15 (24.2) |  |
| 6-10 | 335 (39.2) | 320 (40.4) | 15 (24.2) |  |
| 11-20 | 332 (38.8) | 307 (38.7) | 25 (40.3) |  |
| >20 | 80 (9.4) | 73 (9.2) | 7 (11.3) |  |
| **Site (%)** |  |  |  | 0.373 |
| Gastric | 443 (51.8) | 407 (51.3) | 36 (58.1) |  |
| Non-gastric | 412 (48.2) | 386 (48.7) | 26 (41.9) |  |
| **Treatment duration (years) (%)** | |  |  | 0.274 |
| 0≤t<1 | 195 (22.8) | 181 (22.8) | 14 (22.6) |  |
| 1≤t<2 | 126 (14.7) | 113 (14.2) | 13 (21.0) |  |
| 2≤t<3 | 124 (14.5) | 117 (14.8) | 7 (11.3) |  |
| 3≤t<4 | 248 (29.0) | 235 (29.6) | 13 (21.0) |  |
| 4≤t<5 | 98 (11.5) | 91 (11.5) | 7 (11.3) |  |
| t≥5 | 64 (7.5) | 56 (7.1) | 8 (12.9) |  |

*SD*, Standard Deviation; *HPF*, High-Power Fields

**Table S3.** The analysis of patient clinical characteristics based on mitosis count.

| **Characteristics** | **Overall** | **Mitosis count/50 HPFs** | | | | ***P* value** |
| --- | --- | --- | --- | --- | --- | --- |
|  |  | **≤5** | **6-10** | **11-20** | **>20** |  |
| **n** | 855 | 108 | 335 | 332 | 80 |  |
| **Size (cm) (mean±SD)** | 9.92 (5.59) | 10.69 (4.47) | 10.08 (5.41) | 8.57 (5.30) | 13.85 (6.70) | <0.001 |
| **Size (cm) (%)** |  |  |  |  |  |  |
| 2.0-5.0 | 140 (16.4) | 0 (0.0) | 32 (9.6) | 103 (31.0) | 5 (6.2) |  |
| 5.1-10.0 | 448 (52.4) | 65 (60.2) | 210 (62.7) | 146 (44.0) | 27 (33.8) |  |
| 10.1-15.0 | 138 (16.1) | 30 (27.8) | 49 (14.6) | 46 (13.9) | 13 (16.2) |  |
| >15.0 | 129 (15.1) | 13 (12.0) | 44 (13.1) | 37 (11.1) | 35 (43.8) |  |
| **Ruptured (%)** |  |  |  |  |  | 0.011 |
| Yes | 62 (7.3) | 15 (13.9) | 15 (4.5) | 25 (7.5) | 7 (8.8) |  |
| No | 793 (92.7) | 93 (86.1) | 320 (95.5) | 307 (92.5) | 73 (91.2) |  |
| **Site (%)** |  |  |  |  |  | <0.001 |
| Gastric | 443 (51.8) | 27 (25.0) | 175 (52.2) | 189 (56.9) | 52 (65.0) |  |
| Non-gastric | 412 (48.2) | 81 (75.0) | 160 (47.8) | 143 (43.1) | 28 (35.0) |  |
| **Treatment duration (years) (%)** | |  |  |  |  | 0.074 |
| 0≤t<1 | 195 (22.8) | 36 (33.3) | 73 (21.8) | 70 (21.1) | 16 (20.0) |  |
| 1≤t<2 | 126 (14.7) | 13 (12.0) | 40 (11.9) | 58 (17.5) | 15 (18.8) |  |
| 2≤t<3 | 124 (14.5) | 21 (19.4) | 46 (13.7) | 46 (13.9) | 11 (13.8) |  |
| 3≤t<4 | 248 (29.0) | 20 (18.5) | 106 (31.6) | 103 (31.0) | 19 (23.8) |  |
| 4≤t<5 | 98 (11.5) | 9 (8.3) | 45 (13.4) | 32 (9.6) | 12 (15.0) |  |
| t≥5 | 64 (7.5) | 9 (8.3) | 25 (7.5) | 23 (6.9) | 7 (8.8) |  |

*SD*, Standard Deviation; *HPF*, High-Power Fields

**Table S4.** The analysis of patient clinical characteristics based on tumor site.

| **Characteristics** | **Overall** | **Tumor site** | | **P value** |
| --- | --- | --- | --- | --- |
|  |  | **Gastric** | **Non-gastric** |  |
| **n** | 855 | 443 | 412 |  |
| **Size (cm) (mean±SD)** | 9.92 (5.59) | 10.56 (6.16) | 9.25 (4.81) | 0.001 |
| **Size (cm) (%)** |  |  |  |  |
| 2.0-5.0 | 140 (16.4) | 74 (16.7) | 66 (16.0) |  |
| 5.1-10.0 | 448 (52.4) | 206 (46.5) | 242 (58.7) |  |
| 10.1-15.0 | 138 (16.1) | 83 (18.7) | 55 (13.3) |  |
| >15.0 | 129 (15.1) | 80 (18.1) | 49 (11.9) |  |
| **Mitotic/50 HPFS (mean±SD)** | 13.85 (18.52) | 14.60 (16.44) | 13.05 (20.51) | 0.224 |
| **Mitotic/ 50 HPFs (%)** |  |  |  |  |
| ≤5 | 108 (12.6) | 27 (6.1) | 81 (19.7) |  |
| 6-10 | 335 (39.2) | 175 (39.5) | 160 (38.8) |  |
| 11-20 | 332 (38.8) | 189 (42.7) | 143 (34.7) |  |
| >20 | 80 (9.4) | 52 (11.7) | 28 (6.8) |  |
| **Ruptured (%)** |  |  |  | 0.373 |
| Yes | 62 (7.3) | 36 (8.1) | 26 (6.3) |  |
| No | 793 (92.7) | 407 (91.9) | 386 (93.7) |  |
| **Treatment duration (years) (%)** | |  |  | 0.259 |
| 0≤t<1 | 195 (22.8) | 108 (24.4) | 87 (21.1) |  |
| 1≤t<2 | 126 (14.7) | 71 (16.0) | 55 (13.3) |  |
| 2≤t<3 | 124 (14.5) | 59 (13.3) | 65 (15.8) |  |
| 3≤t<4 | 248 (29.0) | 123 (27.8) | 125 (30.3) |  |
| 4≤t<5 | 98 (11.5) | 55 (12.4) | 43 (10.4) |  |
| t≥5 | 64 (7.5) | 27 (6.1) | 37 (9.0) |  |

*SD*, Standard Deviation; *HPF*, High-Power Fields
